# Chagas disease and perceived quality of life: a cross-sectional study

**DOI:** 10.1590/0037-8682-0206-2023

**Published:** 2023-10-30

**Authors:** Nayara Ragi Baldoni, Nayara Dornela Quintino, Claudia Di Lorenzo Oliveira, José Luiz Padilha da Silva, Ariela Mota Ferreira, Antonio Luiz Pinho Ribeiro, Ester Cerdeira Sabino, Clareci Silva Cardoso

**Affiliations:** 1 Universidade Federal de São João del-Rei, Departamento de Medicina, Divinópolis, MG, Brasil.; 2 Universidade de Itaúna, Itaúna, MG, Brasil.; 3 Universidade Federal do Paraná, Departamento de Estatística, Curitiba, PR, Brasil.; 4 Universidade Estadual de Montes Claros, Programa de Pós-Graduação em Ciências da Saúde, Montes Claros, MG, Brasil.; 5 Universidade Federal de Minas Gerais, Hospital das Clínicas, Faculdade Medicina, Belo Horizonte, MG, Brasil.; 6 Universidade de São Paulo, Instituto de Medicina Tropical, São Paulo, SP, Brasil.

**Keywords:** Chagas disease, Quality of life, Neglected diseases, Blood donor

## Abstract

**Background:**

Chagas disease (ChD) is a neglected tropical disease that is caused by the protozoan parasite *Trypanosoma cruzi* and can negatively impact quality of life (QoL). This study aimed to assess and compare QoL between individuals with and without ChD.

**Methods:**

This cross-sectional study was performed within a concurrent cohort study (REDS). The participants were derived from two blood donation centers: São Paulo capital and Montes Claros, Minas Gerais, Brazil. Participants with ChD were identified in blood donations by serological diagnosis between 2008 and 2010, and those without ChD were donors with negative serology identified during the same period. QoL was assessed using the World Health Organization Quality of Life-BREF questionnaire. Logistic regression was used to compare sociodemographic and clinical characteristics between the groups, and mean, standard deviation, and beta regression were used to compare QoL.

**Results:**

In total, 611 individuals participated in the study (328 with ChD and 283 without ChD). Participants with ChD had lower QoL in the physical (p=0.02) and psychological (p<0.01) domains than did individuals without CD.

**Conclusions:**

Individuals with ChD had worse QoL perceptions. These results provide a comprehensive understanding of the impact of ChD on individuals' QoL, while also highlighting potential opportunities for improving the care and treatment of those affected.

## INTRODUCTION

Chagas disease (ChD) is a neglected tropical disease that is caused by *Trypanosoma cruzi*and is endemic to continental Latin American countries; the highest numbers of infected individuals is found in Argentina (1,505,235), Brazil (1,156,821), and Mexico (876,458), while the highest prevalence is found in Bolivia (607,186 cases/6.1%)^1^ ^,^ ^2^ ^.^ Owing to immigration and globalization, ChD affects individuals in regions other than Latin America, such as North America and Europe^3^ ^.^

Healthcare for individuals with ChD needs to be longitudinal and comprehensive, considering the potential of the disease to affect multiple systems, such as the cardiovascular, digestive, and central nervous systems^2^ ^,^ ^4^ ^.^ Among these complications, cardiovascular disease is the most prevalent and can lead to disability and death^4^ ^,^ ^5^. Approximately 30% of individuals with ChD have cardiac manifestations and manifest worse health outcomes without the necessary care from health services^2^ ^,^ ^5^. However, the access of patients with ChD to adequate medical care remains an important challenge for health systems^6^.

Complications from ChD can lead to death, and it is estimated that 10,000-14,000 patients with ChD die each year worldwide^7^. In addition to mortality, health service costs increase, and quality of life (QoL) is compromised^8^ ^-^ ^10^. In addition to the economic cost and impairment of QoL, ChD mainly affects vulnerable people and communities with limited access to health services, limited educational opportunities, and low income^10^ ^-^ ^14^. Thus, it is of great importance that health policies act on the social determinants of health to contain the social costs of ChD^15^.

Coping strategies for improving the QoL of patients with ChD are rarely used in the context of health services^16^. Notably, for such strategies to be sustainable, they must address the social determinants of health, early diagnosis, and timely treatment^17^. Studies have shown that pharmacological and non-pharmacological treatments and bone marrow cell transplantation effectively control disease evolution and positively affect QoL^18^ ^-^ ^21^. Therefore, evaluating the QoL of these individuals is important for planning ChD coping strategies for subsequent organization^10^. Thus, this study aimed to assess and compare QoL between individuals with and without ChD.

## METHODS

The cross-sectional study was conducted within a follow-up cohort from the “Retrovirus Epidemiology Donor Study-II (REDS-II),” which has been previously described in detail^22^ ^-^ ^24^. Participants were recruited from a list of blood donors from the Hemominas Foundation (Montes Claros, MG, Brazil and the Pró-Sangue Foundation (São Paulo, SP) from July 2008 to October 2010. Donors who tested positive for *T. cruzi* were considered to have the disease, and donors who were negative were considered to be without the disease; the two groups were matched according to sex, age group, and blood center location. 

During the first visit, participants underwent serology, electrocardiogram (ECG), and echocardiography tests and answered a questionnaire that included clinical and sociodemographic variables. To validate a diagnosis of Chagas cardiomyopathy, participants underwent expert evaluation (trigger)^22^. 

Subsequently, at the second visit between 2018 and 2019, 10 years later, participants were re-evaluated. At this stage, demographic, clinical, lifestyle, health condition, disease treatment, and QoL information were collected. To assess QoL, the World Health Organization Quality of Life (WHOQOL)-BREF instrument was used; this instrument has been validated in Brazil, and the results showed that the characteristics were satisfactory in terms of internal consistency, discriminant validity, criterion validity, concurrent validity, and test-retest reliability. Furthermore, the WHOQOL-BREF has demonstrated adequate psychometric performance for validity and reliability in the Brazilian population^25^.

The WHOQOL-BREF comprises 26 questions: two general QoL questions and 24 questions distributed across four domains: physical, psychological, social relations, and environment. QoL was assessed using a five-point Likert-type scale, where a higher score indicates a better the perception of QoL in the last 15 days. The inclusion criteria for QoL assessment were the ability to understand the questions of the instrument and conduct the self-assessment. In this study, five interviewers conducted the interviews; these interviewers were previously trained to achieve 80% inter-rater agreement when applying the scales, using a senior interviewer as a reference.

The dependent variable in this study was QoL. The independent variables were sociodemographic (age, income, number of people in the household, sex, skin color, knowledge of how to read and write, and education), behavioral (smoking, physical activity, and alcoholic beverage consumption), and clinical (presence of comorbidities, ECG changes, and echocardiogram changes) characteristics. The clinical variable, presence of cardiomyopathy, was classified using the following criteria: left ventricular ejection fraction <50% and/or QRS interval ≥120 ms^24^ ^,^ ^26^.

### Statistical Analysis

Sociodemographic, behavioral, and clinical variables are summarized using means, standard deviations, and absolute and relative frequencies. Violin plots were used to depict the distribution of QoL among the ChD groups. To assess QoL, the results for each domain were transformed into a linear scale ranging from 0 to 100. Unadjusted comparisons between cases and controls were performed using logistic regression models. Sociodemographic, behavioral, and clinical variables were entered as predictors in the univariate models, while the Chagas score was the response variable.

To compare QoL domains, univariate and multivariate inflated beta regression models belonging to the Generalized Additive Models for Location, Scale and Shape (GAMLSS) were constructed^27^. The beta regression model is suitable for modelling outcome variables measured on a percentage scale. Wald-type and Likelihood Ratio tests were performed. The logit link function was adopted for all models, in which the exponential of the regression parameters was interpreted in terms of the odds ratio (OR). Therefore, an OR<1 indicates a lower QoL, whereas an OR>1 indicates better QoL. Multivariate models for QoL included explanatory variables that were statistically significant in the exploratory analysis and those with clinical relevance. Multicollinearity was tested, and highly correlated variables were not included in the models. 

The goodness-of-fit model was evaluated using quantile residual plots, Q-Q plots, and summary statistics. Multiple imputations were used to assess differential bias due to 155 missing values for monthly family income. The predictive mean-matching imputation model was used to impute missing monthly family income values conditional on disease group, age, education, donation center, ejection fraction, and the four QoL domains. Additionally, a single missing value in the ejection fraction was imputed using the same configuration. The results of 50 multiply imputed datasets were combined using Rubin’s rules. The significance level was set at 5% for all analyses. For the analyses, R software version 4.2.2 was used with the packages *haven* (data importing), *tidyverse* (data manipulation), *ggplot2* (graphs), *gamlss* (model fitting), and *mice* (multiple imputation).

### Ethical Considerations

This project was approved by the Research Ethics Committee of the University of São Paulo under opinion numbers 6023 and CAAE:00580612.8.0000.0065 on March 7^th^, 2012. This study was also approved by the Brazilian National Ethics Committee (CONEP No. 1312/2006) on December 20^th^, 2012.

## RESULTS

**FIGURE 1:**
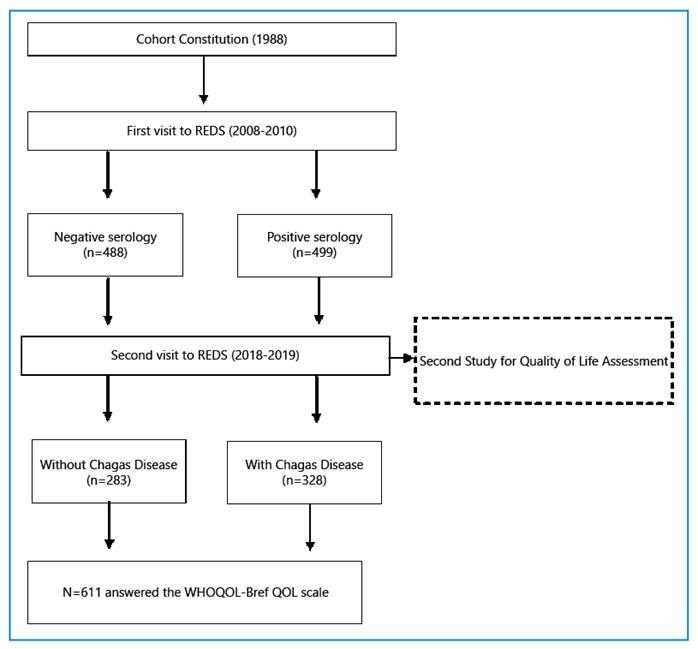
Flowchart of the study design and constitution of the groups.

A total of 611 participants responded to the interview (328 with ChD and 283 without ChD). The population selection flowchart is shown in Figure 1.

The sociodemographic, behavioral, and clinical characteristics of the participants are presented in Table 1. Among all participants, a majority (51.8% with ChD and 50.9% without ChD) were women. The mean age for participants with and without ChD was 56.2 (±9.9) years and 58.4 (±9.6) years, respectively. Mean monthly income was US$22.97 (±15.49) vs. US$32.07 (±22.37) (p<0.001). In addition, the educational level and percentage of participants who “know how to read and write” (79.6% vs. 92.6%, p<.0.001) was lower in participants with ChD. Most participants in both groups were black/mixed (64.4% vs. 54.1%, p=0.034) (Table 1). 

**TABLE 1:** Sociodemographic, behavioral, and clinical characteristics of participants with and without Chagas disease (ChD) 2018-2019.

Variables	Without ChD (n=283)	With ChD (n=328)	p-value*
Sociodemographic			
Age	58.4 (±9.6)	56.2 (±9.9)	0.008
Family income (Mean, SD**)	32.07 (±22.37)	22.97 (±15.49)	<0.001
*No. of people in the household*			
<4	183/283 (64.7)	204/328 (62.2)	0.528
≥4	100/283 (35.3)	124/328 (37.8)	
*Sex*			
Male	139/283 (49.1)	158/328 (48.2)	0.816
Female	144/283 (50.9)	170/328 (51.8)	
*Skin color*			
Black/Mixed	153/283 (54.1)	210/326 (64.4)	0.034
White	119/283 (42.1)	107/326 (32.8)	
Other^a^	11/283 (3.9)	9/326 (2.8)	
*Can read and write*			
No	21/283 (7.4)	67/328 (20.4)	<0.001
Yes	262/283 (92.6)	262/328 (79.6)	
*Education*			
Illiterate	6/283 (2.1)	32/328 (9.8)	<0.001
1 to 9 years	119/283 (42.1)	182/328 (55.5)	
9 to 12 years	100/283 (35.3)	101/328 (30.8)	
>12 years	58/283 (20.5)	13/328 (3.9)	
**Behavioral**			
*Smoking*			
never smoked	179/282 (63.5)	228/328 (69.5)	0.282
have smoked in the past	79/282 (28.0)	78/328 (23.8)	
currently smokes	24/282 (8.5)	22/328 (6.7)	
*Physical activity* ^*b*^			
No	149/283 (52.7)	179/328 (54.6)	0.635
Yes	134/283 (47.3)	149/328 (45.4)	
*Alcoholic beverages* ^*c*^			
No	159/277 (57.4)	212/323 (65.6)	0.039
Yes	118/277 (42.6)	111/323 (34.4)	
**Clinical**			
*Diabetes mellitus*			
No	245/281 (86.5)	278/320 (86.9)	0.886
Yes	38/281 (13.5)	42/320 (13.1)	
*SAH* ^*d*^			
No	171/271 (63.1)	192/319 (60.2)	0.469
Yes	100/271 (36.9)	127/319 (39.8)	
*Thyroid*			
No	244/276 (88.4)	286/318 (89.9)	0.549
Yes	32/276 (11.6)	32/318 (10.1)	
*Renal*			
No	263/278 (94.6)	299/320 (93.4)	0.550
Yes	15/278 (5.4)	21/320 (6.6)	
*AMI* ^*e*^			
No	274/283 (96.8)	313/324 (96.6)	0.882
Yes	9/283 (3.2)	11/324 (3.4)	
*Coagulopathies*			
No	265/276 (96.0)	298/313 (95.2)	0.635
Yes	11/276 (4.0)	15/313 (4.8)	
*Cardiomyopathy*			
No	269/283 (95.1)	231/326 (70.9)	<0.001
Yes	14/283 (4.9)	95/326 (29.1)	
ECG Summary Measure***			
Normal	63/283 (22.3)	59/328 (18.0)	<0.001
Smaller	156/283 (55.1)	139/328 (42.4)	
Larger	64/283 (22.6)	130/328 (39.6)	
*Left ventricular ejection fraction*			
Normal > 50%	277/283 (97.9)	302/327 (92.4)	0.004
Abnormal < 50%	6/283 (2.1)	25/327 (7.6)	
*QRS*			
<120 ms	273/285 (96.5)	237/327 (72.5)	<0.001
≥120 ms	10/285 (3.5)	90/327 (27.5)	

* p-values from univariate logistic models; **SD: standard deviation; Income: every 100 units; ***ECG: electrocardiogram. ^a^Others: Asian (with Chagas: 8/326; without Chagas: 11/283) and indigenous (with Chagas: 1/326; without Chagas: 0/283). ^b^Physical activity refers to some activity or sport (walking, soccer, swimming, running, etc.).^c^alcohol use in the last 30 days. ^d^systemic arterial hypertension. ^e^acute myocardial infarction

A majority in both groups did not engage in physical activity (54.6% with ChD and 52.7% without ChD) or drink alcohol in the days prior to answering the questionnaire (65.6% and 57.4%, respectively). Moreover, cardiomyopathy was significantly (p<0.001) more frequent in participants with ChD (29.1% vs. 4.9%), as were major ECG alterations (39.6% vs. 22.6%; p<0.001). The main comorbidities among both groups were systemic arterial hypertension (39.8% and 36.9%, respectively) and diabetes mellitus (13.1% and 13.5%, respectively) (Table 1). The use of polypharmacy (use of ≥5 medications) was 2.5% and 2.1%, respectively.

**FIGURE 2:**
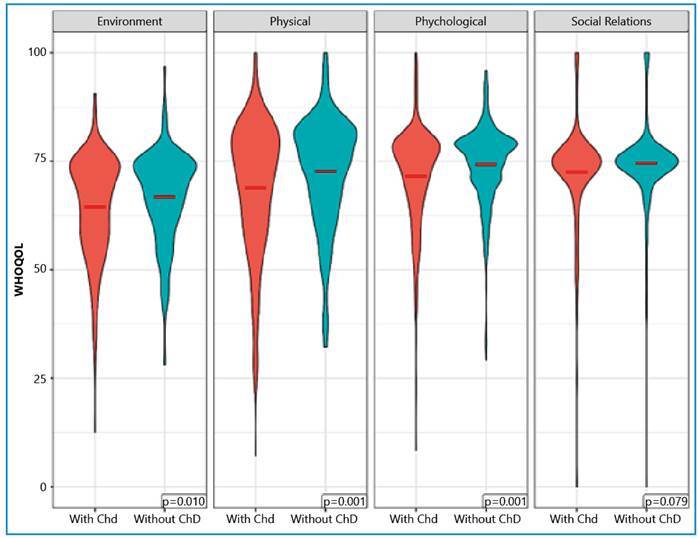
Violin plot of quality of life as assessed using the WHOQOL-BREF domain in participants with and without Chagas disease (ChD).

QoL assessment results are shown in Table 2. All QoL domains of participants with ChD had a lower mean when compared to those without ChD. The most compromised QoL domain was the environment in both groups, with means of 64.52 and 66.8 for participants with and without ChD, respectively. For all OR values <1, participants with ChD had a lower QoL than those without ChD (reference). Without controlling for confounders, a significant difference was observed in three of the four domains evaluated: physical (p=0.001), psychological (p=0.001), and environmental (p=0.010). In the multivariate models, after multiple imputations, statistically significant differences were observed in the physical (p=0.018) and psychological (p=0.007) domains. Figure 2 shows the violin plots of the distribution of QoL by domain between the two groups. 

**TABLE 2:** Results of quality of life domains as assessed using the WHOQOL-BREF in participants with and without Chagas disease (ChD).

Domain	With ChD (n=328)	Without ChD (n=283)	OR (95% CI)^a^	OR (95% CI)^b^	OR (95% CI)^c^
	Mean (SD)	Mean (SD)			
Physical	68.86 (±15.24)	72.66 (±12.42)	0.85 (0.76 - 0.93); p=0.001	0.87 (0.77 - 0.98); p=0.025	0.88 (0.79 - 0.98); p=0.018
Psychological	71.57 (±11.44)	74.25 (±9.36)	0.87 (0.81 - 0.95); p=0.001	0.86 (0.77 - 0.95); p=0.004	0.88 (0.79 - 0.98); p=0.007
Social Relations	72.51 (±12.55)	74.55 (±9.85)	0.95 (0.89 - 1.01); p=0.079	0.95 (0.89 - 1.01); p=0.188	0.96 (0.90 - 1.03); p=0.290
Environment	64.52 (±11.58)	66.8 (±10.30)	0.90 (0.84 - 0.98); p=0.010	0.93 (0.85 - 1.02); p=0.110	0.95 (0.87 - 1.03); p=0.185

^a^Beta regression: not adjusted. ^b^Beta regression: adjusted for age, family income, skin color, years of education, blood center, and ejection fraction. ^c^Beta regression: multiple imputations adjusted for the same variables as in (b). **OR:** odds ratio; **CI:** confidence interval. Domain scores range from 0 to 100.

## DISCUSSION

This study revealed that participants with ChD had a lower QoL in the physical and psychological domains than those without ChD. These differences can be explained by the clinical characteristics of individuals with ChD, who exhibited a higher prevalence of cardiomyopathy and other chronic diseases. The current findings corroborate those of other investigations concerning QoL assessment in patients with ChD. One study also used the WHOQOL-BREF and found that the physical domain was the most compromised^28^. Similar to the present study, another study used different instruments to compare QoL (Minnesota Living with Heart Failure Questionnaire and Short-Form-36) reported significantly worse QoL scores for the Physical and Role-Emotional domains in the group with ChD compared to the group without CHD^29^. Thus, these results can be useful for planning care strategies for individuals with ChD, focusing on the aspects of daily life assessed in this domain.

A potential explanation for the lower QoL perception in the physical domain among patients with ChD could be linked to a low ejection fraction and higher QRS interval, which indicate cardiomyopathy. However, it is noteworthy that we could not determine the extent of heart disease in these patients, as our investigation assessed only the presence or absence of cardiomyopathy. ChD is a chronic, neglected condition that affects a population with a lower income and that resides in remote locations or rural areas^13^ ^,^ ^12^ ^,^ ^30^. These factors can lead to greater difficulty in accessing health services and obtaining specialized care such as would be provided by a cardiologist. These factors lead to worsening of the disease and consequently reflect the low QoL of these individuals. In a 2018 study, Santos-Filho et al. observed that participants with the cardiodigestive form of ChD, including heart failure, had lower QoL scores than those with other clinical forms of ChD^28^.

Numerous barriers exist regarding access to ChD diagnosis and treatment^31^. The diagnosis of *T. cruzi* infection and timely follow-up capable of identifying the onset or progression of cardiomyopathy can mitigate morbidity, improve survival, and positively impact QoL^32^. Therefore, we emphasize the importance of training health professionals in the general knowledge of ChD, including screening, diagnosis, and adequate treatment^33^. Early diagnosis and timely treatment, in addition to preventing disease worsening, can reduce healthcare costs.

In addition to the economic impact on the health system, the cost of treating ChD is substantial and further results in a decrease in QoL; the World Health Organization emphasizes decreased productivity due to premature death and absenteeism of affected workers^34^. Considering the significant impact of the three pillars, namely high costs for the health system, decreased quality of life, and reduced productivity on both individuals and the economy, it is crucial for healthcare managers to prioritize investment in case-tracking strategies. Early diagnosis is key to timely ChD treatment, which would help mitigate the negative consequences.

Participants with ChD manifested a worse perception of QoL in the environment domain compared to those without ChD. This finding may be related to the higher family incomes of these individuals. The environment domain assesses aspects related to physical safety, physical environment, financial resources, new information/skills, recreation and leisure, home environment, healthcare, and transportation. Thus, a higher income may indicate greater access to these aspects. 

Moreover, the current study population comprised participants from two municipalities of varying sizes located in the same southeastern region of the country, where no specialized Chagas center is available. Consequently, limited access to healthcare services, along with other factors such as transportation and social assistance, may prevent participants from receiving comprehensive care for ChD and other conditions. In addition, participants who reside in the capital, São Paulo, may be more vulnerable owing to physical security and greater air and noise pollution. In contrast, for participants from Montes Claros (MG), the problems previously presented were not part of their routine, but social, recreational, and leisure activities were limited.

Another important finding was the sociodemographic characteristics of participants. Scientific findings of lower levels of education and poverty in individuals with ChD are common^11^. Although the study population was recruited from blood centers, the findings did not differ from those in the literature. Participants with ChD had less education, lower income, and a lower ability (20%) to read or write. An awareness that these characteristics are common in individuals with ChD demands simple communication from health professionals so that the patient understands the information and can follow the prescribed recommendations and treatments. Therefore, it is of great importance to assess health literacy to determine the self-care capacity of individuals with ChD. As evidenced by a 2020 study by Quintino et al., patients with ChD with low health literacy had worse QoL scores and unfavorable clinical outcomes^11^.

The aim of this study was to show that ChD negatively impacted QoL. If used in health management, this information can be useful in organizing the care of patients with ChD. As a limitation, we can point out information bias, considering that only participants who were able to answer the WHOQOL-BREF participated in the QoL assessment. This may have influenced the assessed QoL, as individuals with a worse state of health were not able to answer the instrument with a self-assessment of QoL. Another limitation is that the patients were not assessed for the presence of digestive complications, which can also compromise QoL.

## CONCLUSION

In view of these findings, it is evident that individuals with ChD had a worse perception of QoL than those without ChD, demonstrating that ChD negatively impacted QoL. This further demonstrates that access to essential services may affect positive perceptions of QoL. Therefore, to reduce complications or negative impacts on perceived QoL in patients with ChD, improved healthcare, such as screening, early diagnosis, and timely treatment, are needed; furthermore, greater investments are needed in health education programs and preventive care, especially in endemic areas.
